# Acute ischaemic stroke in *Listeria monocytogenes* meningoencephalitis

**DOI:** 10.1259/bjrcr.20190068

**Published:** 2020-02-12

**Authors:** Surrin S. Deen, Jennifer Boyes, Bankole Oyewole, Anna Bahk, George Thomas, Gunaratnam Gunathilagan

**Affiliations:** 1 Department of Radiology, University of Cambridge, Cambridge, United Kingdom; 2 East Kest Hospitals University NHS Trust, Margate, United Kingdom; 3 School of Medical Education, King's College London, London, United Kingdom

## Abstract

*Listeria monocytogenes* is the third most frequent cause of bacterial meningitis and has a predilection for elderly patients and the immunosuppressed. A small number of patients with *Listeria monocytogenes* meningoencephalitis have previously been reported to experience stroke-like symptoms that were attributed to microabscess formation and the mass effect of collections of infection in the brain. These infections led to temporary neurological deficits that resolved with antimicrobial treatment, rather than to true strokes with permanent neurological deficits. This report discusses the case of an 80- year-old male, who was immunosuppressed with mesalazine for the treatment of Crohn’s disease, and who went on to develop *Listeria monocytogenes* meningoencephalitis. 1 week into his admission, for antibiotic therapy, the patient began to experience new onset right upper limb weakness, nystagmus and past pointing. These symptoms were initially thought to be a complication of the infection. However, subsequent diffusion-weighted MRI revealed that the patient had more likely suffered an acute ischaemic event and a contrast-enhanced MRI performed later could not detect any abscess or large infective focus in a region that could explain the symptoms. This case report highlights the fact that ischaemic and infective pathologists may coexist in immunosuppressed Listeria patients and that clinical signs and symptoms should guide the use of appropriate imaging modalities such as MRI to clarify differentials so that ischaemia is not mistaken for the more common stroke mimic caused by infection in these patients.

## Clinical presentation

An 80-year-old male presented to the emergency department with fever and confusion. He had a background of Crohn’s disease, diabetes mellitus type II, atrial fibrillation and hypertension. For these conditions, he took mesalazine 1.5 g three times a day, insulin injections, bisoprolol 1.25 mg once daily and rivaroxaban 20 mg once daily. He was assessed by the emergency physicians and found to have pain on neck flexion. Blood tests revealed raised serum C-reactive protein and a CT of the head showed age-related changes but no acute abnormalities. Blood cultures were taken and a lumbar puncture was performed for cerebrospinal fluid sampling. The patient then began empirical treatment for a presumed diagnosis of bacterial meningitis and his mesalazine use was put on hold. Cerebrospinal fluid polymerase chain reaction analysis later revealed the presence of *Listeria monocytogenes* and after discussion with the hospital microbiologist, antibiotic treatment was changed to a 6-week course of intravenous amoxicillin. 2 days later, the patient’s blood culture results also confirmed *Listeria monocytogenes* growth. The features of meningism began to gradually improve with amoxicillin treatment but on day 7 of admission, the patient developed new right upper limb numbness, nystagmus and past pointing on the right side.

## Differential diagnosis

Initially, it was thought that the most likely cause for the patient’s new neurological symptoms was progression of his infection. This suspicion was based off previous literature describing abscess formation as the most common source of stroke-like symptoms in immunosuppressed *Listeria monocytogenes* meningoencephalitis patients.^1–4^ A true stroke was also considered as a possibility at this point, but ischaemia was felt to be less likely due to the patient’s anticoagulation with rivaroxaban and a lack of any reports that the clinical team could identify describing an ischaemic stroke occurring simultaneously with active Listeria meningoencephalitis infection. Imaging was nevertheless performed to assess for progression of infection, ischaemic or haemorrhagic stroke

## Investigation/Imaging findings

**Figure 1.**
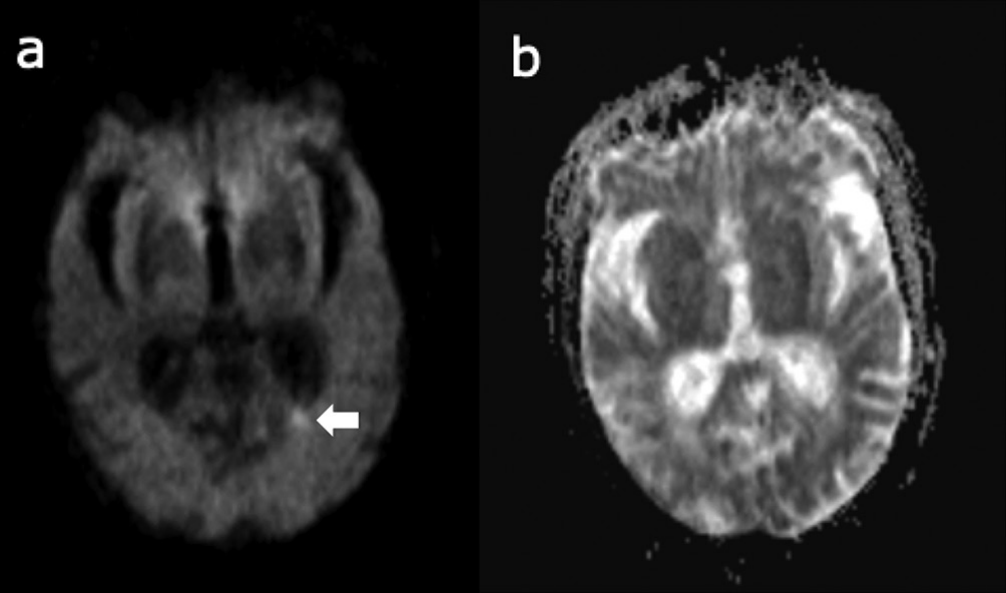
(a) DWI showing a small left occipital lesion (arrow); (b) ADC map does not depict a clear lesion corresponding to that seen on DWI. ADC, apparent diffusion coefficient; DWI, diffusion-weightedimaging.

CT of the head after the development of limb numbness, nystagmus and past pointing, showed no changes compared to the CT imaging from 1 week earlier at admission. MRI of the head was therefore performed, but diagnostic quality was limited due to agitation and movement by the patient, diffusion-weighted imaging (DWI) however revealed a small hyperintensity in the left occipital lobe white matter adjacent to the lateral ventricle. This is shown in Figure 1.

**Figure 2.**
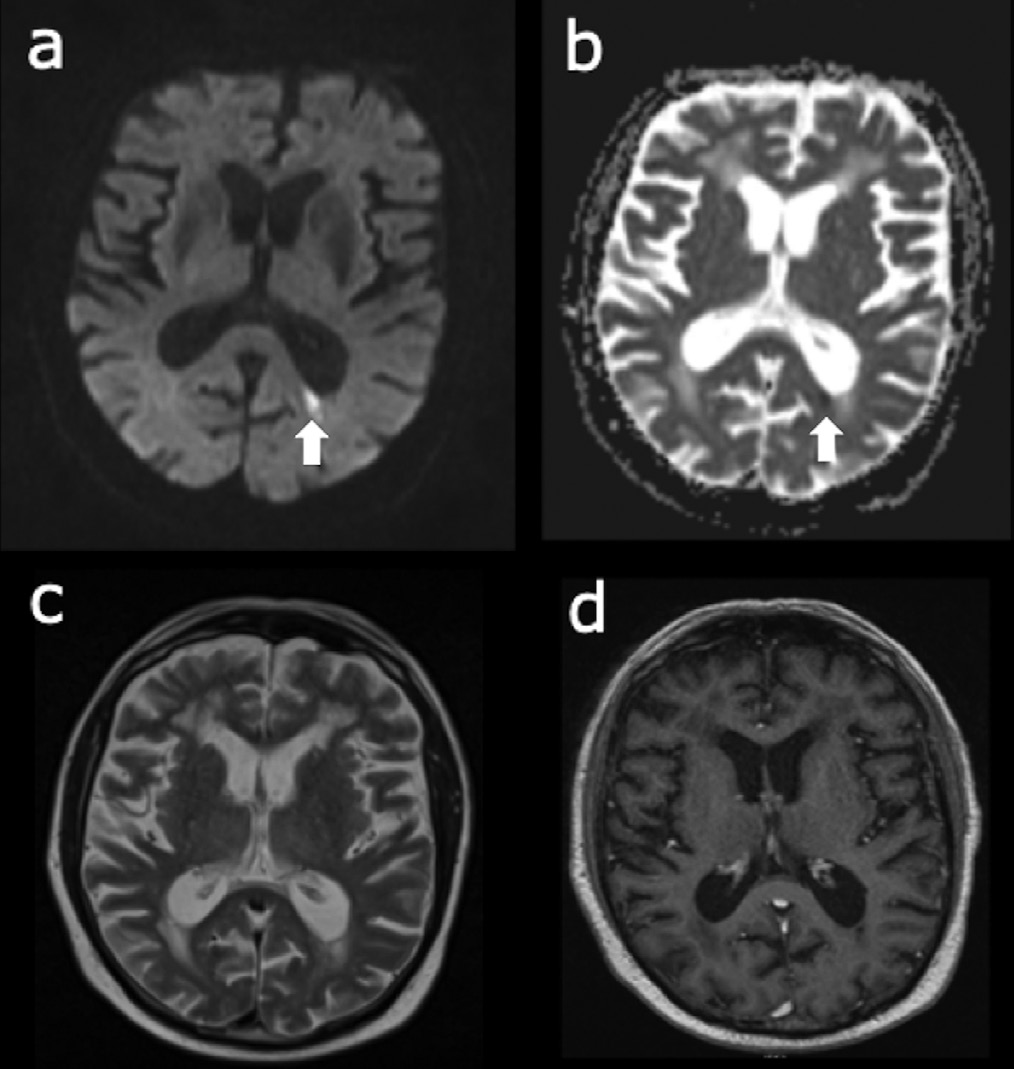
Repeat MRI images under sedation (a) DWI image showing a small left occipital lesion (arrow); (b) ADC map showing a small area of low ADC (arrow) corresponding to the lesion on DWI; (c) *T*_2_ weighted image showing no significant changes in the region of the lesion; (d) post-contrast image showing no contrast uptake or ring-enhancement at the site of the lesion. ADC, apparent diffusion coefficient; DWI, diffusion-weighted imaging.

After detection of this small occipital lesion, a repeat MRI was performed under mild benzodiazepine sedation and with contrast, which showed high signal in the dependent regions within each lateral ventricle, likely representing ventriculitis related to the Listeria meningoencephalitis. The small focus of high DWI signal was again seen in the left occipital lobe, adjacent to the medial aspect of the occipital horn of the left lateral ventricle. On the apparent diffusion coefficient map, this lesion was of low signal, in keeping with a small focal infarct in the area. There was a normal craniocervical junction region, moderate generalized atrophy and severe periventricular and corona radiata deep white matter microangiopathic changes. There was no convincing focus of intracranial haemorrhage and nothing to suggest haemorrhagic transformation of the small left occipital infarct. Post-contrast, there were no signs of intracranial abscess or empyema and no evidence of a space-occupying lesion. The dural venous sinuses were patent and there were no significant findings identified involving the arterial tree at the level of the circle of Willis. The MRI slices showing the occipital lesion are displayed in Figure 2.

**Figure 3.**
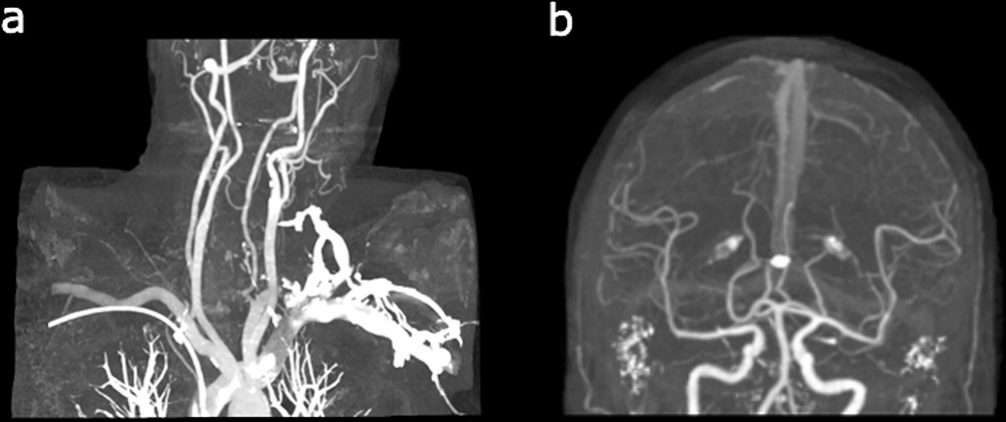
The patient’s CT angiogram of (a) the carotids showing no significant stenosis and (b) the intracranial blood vessels.

CT angiogram of the carotids was performed after the MRI confirming stroke but did not find any source of emboli in the carotids. There was no significant stenosis, aneurysm or occlusion detected in any major blood vessel, although some calcification was noted at the proximal internal carotid artery on the left and within the distal vertebral artery on the right. The appearance of the CT angiogram is shown in Figure 3.

### Treatment, outcome and follow-up

The patient’s stroke was treated by pausing rivaroxaban and initiating anti platelet therapy with 300 mg of aspirin once daily. After 2 weeks, he was restarted on rivaroxaban and the aspirin was permanently stopped. He spent a total of 38 days in hospital and during this time he also underwent stroke physiotherapy treatment. He was then discharged home and had a follow-up appointment with a stroke consultant in an outpatient clinic three months later. At the follow-up clinic, he still had some residual stroke symptoms that affected his mobility and acted as further confirmation of a true ischaemic event due to the symptom persistence at this time. He required the assistance of Zimmer frame to mobilize and had a Tinetti balance score of 19/28, indicating he now had a moderate falls risk. He also required support from arm rests and rails to facilitate his transfers but was independent with his self-care, including dressing and washing. He also reported that his Crohn’s disease was not fully controlled at the time of follow-up but was due to see a gastroenterologist for adjustment of this treatment.

## Discussion

*Listeria monocytogenes* meningoencephalitis has a higher prevalence in the elderly and in patients who are immunocompromised.^5,6^ Particularly, in the immunosuppressed there can be sufficient accumulation of bacteria to create an abscess that has a mass effect on the CNS and causes focal neurological signs and symptoms.^7,8^ When abscess formation occurs, eradication of the organism becomes more difficult. Early recognition and treatment of *Listeria monocytogenes* meningoencephalitis is therefore important to avoid progression in the immunosuppressed.

The presentation of progressive *Listeria monocytogenes* meningoencephalitis can mimic stroke and cases of stroke mimics have been reported in immunosuppression caused by chronic lymphocytic leukaemia and chronic lymphocytic leukaemia treatment,^2^ in lung cancer with chemotherapy treatment,^3^ in a rheumatoid arthritis patient taking methotrexate and prednisolone^4^ and in a patient taking immunosuppressive therapy for Horton’s arthritis.^1^

The authors of this case report have not been able to identify a published example of stroke symptoms developing in any *Listeria monocytogenes* meningoencephalitis patient, immunosuppressed for Crohn’s, or to identify any other report of a true ischaemic stroke occurring during an episode of active *Listeria monocytogenes* meningoencephalitis infection. Nevertheless, in the case presented here a histological diagnosis of stroke was not established and previous studies have shown that ischemic complications of CNS infection may be associated with vasculitic thrombotic infarction, such as commonly occurs in tuberculosis^9^ or rarely in other infections.^10^ A cardioembolic source for the infarction was also unsupported by any bacteremia or known valvular disease and the possibility of early microabscess formation cannot be completely excluded based on the imaging and clinical findings presented here alone. This patient, however, represents an example of the clinical dilemmas faced when concomitant episodes suggestive of acute cerebral infarct and *Listeria monocytogenes* meningoencephalitis occur and demonstrates how the combination of diffusion weighted and contrast enhanced MRI can be used in an attempt to clarify the diagnoses.

The ability to differentiate neurological deficits caused by infection from those caused by ischaemia is clinically important as the treatments for each condition is different and delays in diagnosis, especially of ischaemic stroke, are associated with higher morbidity. The appearance of ischaemic stroke on DWI depends on timing after the event. Following a stroke there is an initial hyperintensity on DWI and then a decrease in signal towards the later phases. In CNS infection there can be a centrally located restriction in diffusion and more significant restriction of diffusion peripherally if there is an abscess wall present. With contrast-enhanced MRI on the other hand, infarcted nervous tissue does not typically show enhancement; however, infection can demonstrate enhancement both centrally and peripherally in the form of an outer ring.

Cases like this should serve as a reminder to clinicians to consider the possibility of two simultaneous and different pathophysiologies, resulting in symptoms that could otherwise be attributed to only one disease. The more common scenario is for *Listeria monocytogenes* meningoencephalitis to cause a stroke mimic but the possibility of a true stroke should never be overlooked as stroke is a condition which, if missed or incorrectly attributed to a mimic, could result in severe disability or even death. In the future, the early use of diffusion-weighted and contrast-enhanced MRI, when CT head results are non-diagnostic, should always be considered in *Listeria monocytogenes* meningoencephalitis patients who develop stroke-like symptoms including those who are immunosuppressed.

## Learning points

1. Stroke-like symptoms in *Listeria monocytogenes* meningoencephalitis may present in immunocompromised patients due to progression of infection and the mass effect of abscess formation.
2. It is also possible for stroke-like symptoms presenting in immunosuppressed *Listeria monocytogenes* meningoencephalitis patients to represent true cerebral infarct.
3. Diffusion-weighted MRI and contrast-enhanced MRI can help differentiate infective from ischaemic causes of neurological deficit in *Listeria monocytogenes* meningoencephalitis patients and should be considered early in the clinical work-up.
